# Impacts of Green Synthesis Process on Asymmetric Hybrid PDMS Membrane for Efficient CO_2_/N_2_ Separation

**DOI:** 10.3390/membranes11010059

**Published:** 2021-01-15

**Authors:** Guo-Liang Zhuang, Chao-Fong Wu, Ming-Yen Wey, Hui-Hsin Tseng

**Affiliations:** 1 Department of Environmental Engineering, National Chung Hsing University, Taichung 402, Taiwan; d102063002@smail.nchu.edu.tw (G.-L.Z.); jeff@dragonsteel.com.tw (C.-F.W.); 2 School of Occupational Safety and Health, Chung Shan Medical University, Taichung 402, Taiwan; 3 Department of Occupational Medicine, Chung Shan Medical University Hospital, Taichung 402, Taiwan

**Keywords:** gas separations, carbon dioxide capture, water-in-emulsion method, curing temperature

## Abstract

The effects of green processes in hybrid polydimethylsiloxane (PDMS) membranes on CO_2_ separation have received little attention to date. The effective CO_2_ separation of the membranes is believed to be controlled by the reaction and curing process. In this study, hybrid PDMS membranes were fabricated on ceramic substrates using the water-in-emulsion method and evaluated for their gas transport properties. The effects of the tetraethylorthosilicate (TEOS) concentration and curing temperature on the morphology and CO_2_ separation performance were investigated. The viscosity measurement showed that, at specific reaction times, it is benefit beneficial to fabricate the symmetric hybrid PDMS membranes with a uniform and dense selective layer on the substrate. Moreover, the a high TEOS concentration can decrease the reaction time and obtain create the a fully crosslinked structure, allowing more efficient CO_2_/N_2_ separation. The separation performance was furtherly improved with in the membrane prepared at a high curing temperature of 120 °C. The developed membrane shows excellent CO_2_/N_2_ separation with a CO_2_ permeance of 27.7 ± 1.3 GPU and a CO_2_/N_2_ selectivity of 10.3 ± 0.3. Moreover, the membrane shows a stable gas separation performance of up to 5 bar of pressure.

## 1. Introduction

The membrane-based gas separation processes have been proposed to capture CO_2_ due to its many advantages, e.g., energy efficiency, inexpensive, and simplicity, compared to conventional technologies [1]. Tremendous progress has been made in the CO_2_ separation of membranes through the material selection and fabrication processes. The performance of a membrane depends on the material having CO_2_ affinity and on controlling the molecular structure of the membrane. 

Among membrane materials, polydimethylsiloxane (PDMS) rubber is extremely attractive for its exceptional physical/chemical properties. This organosilicon rubber consists of Si–O backbones as its main chain and organic side chains, showing a flexible polymer backbone under a low glass transition temperature (−123 °C) [2]; hence, it possesses high CO_2_ permeability through its high solution-diffusion ability [3]. However, PDMS membranes possess a low CO_2_/N_2_ selectivity of approximately 6.3–9.5 [4,5,6,7] due to their poor size-sieving ability. 

To improve the separation performance of membranes, it has been suggested that different organic or inorganic materials, such as fluorescent material (TPE) [8], metal nanoparticles [9], graphene oxide (GO) [10], carbon nanotubes (CNTs) [11], metal–organic frameworks (MOFs) [12], TiO_2_ [13,14] and silica [15,16] are incorporated. Compared with other materials, silica is a common and low-cost functional filler for organic–inorganic hybrid membranes. Ataeivarjovi et al. [17] verified that the overall mass transfer resistance can be reduced by 49% after the incorporation of silica, resulting in effective gas transport properties. Rosli et al. [18] reported that composite membranes containing hybrid PDMS–silica nanoparticles showed an enhancement in their CO_2_/N_2_ selectivity of 8.39 when compared to the original membrane. Moreover, the incorporation of silica can improve the thermal and mechanical properties of membranes [19,20].

**Scheme 1:**
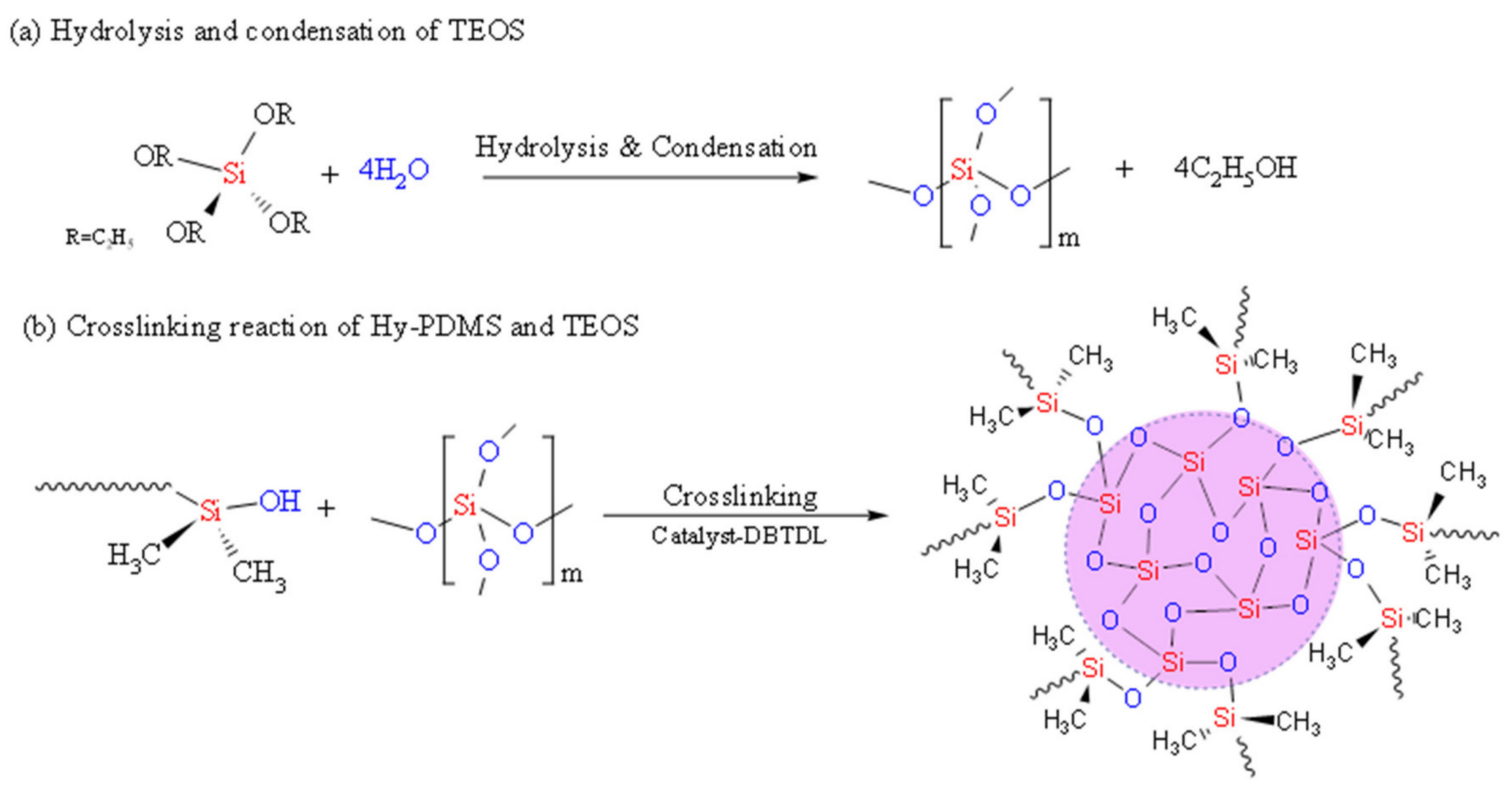
(**a**) Hydrolysis–condensation reaction of tetraethylorthosilicate (TEOS). (**b**) Crosslinking reaction of a hybrid polydimethylsiloxane (PDMS) membranes.

Recently, the green alternative synthetic pathways of organic–inorganic hybrid membrane manufacturing have become a focus for various studies. Hybrid PDMS membranes can be fabricated through a green sol–gel process in water emulsion. This concept was developed by Li [21] and has been demonstrated in previous studies [22,23]. Water-based emulsions contain only trace surfactants, such as 4-Dodecylbenzenesulfonic acid (DBSA) [24,25] and sodium dodecyl sulfate (SDS) [26]. Amphiphilic molecules can help to enhance the synthetic reaction of PDMS in an aqueous solvent by increasing the interaction between the oil(polymer)/water phase. The alternative synthetic pathway is a safe, low-pollution and economically competitive pathway [27,28]. Generally, tetraethylorthosilicate (TEOS) is used as the precursor and crosslinker for forming a siloxane network between PDMS chains in membranes via a crosslinking reaction under the hydrolysis–condensation mechanism [29,30,31]. The green synthesis of hybrid PDMS membranes is shown in Scheme 1. The siloxane network is formed by the hydrolysis–condensation of TEOS in water. Meanwhile, a PDMS/siloxane hybrid is produced by the crosslinking reaction of the siloxane network and the hydroxyl-terminated polydimethylsiloxane (Hy-PDMS). The crosslinking conditions of PDMS membranes is a key contributing factor for gaining excellent structure stability and separation performance, such as the loading of the crosslinker [32] and curing process (time and temperature) [33].

Thus far, there are limited works that have been conducted to study the effect of green synthesis process on the properties of CO_2_ separation. Therefore, the present work focused on the development of hybrid PDMS membranes via green synthesis for CO_2_ separation applications. The effects of the synthesis conditions on the morphology properties and separation performance were investigated. The performance of the as-prepared membrane was then compared to that in the previous literature. As far as the authors know, this is the first report on the fabrication of asymmetric hybrid PDMS membranes using the green synthesis process for CO_2_ separation.

## 2. Materials and Methods 

### 2.1. Materials

Hydroxyl-terminated polydimethylsiloxane (Hy-PDMS), tetraethylorthosilicate (TEOS; purity > 98%), and 4-dodecylbenzenesulfonic acid (DBSA) were purchased from Sigma Aldrich Chemical (USA). Hy-PDMS had a viscosity of 90–120 cPa (average Mw = ~4200). Dibutyltin dilaurate (DBTDL) was of analytical reagent grade and purchased from Alfa Aesar. n-hexane was supplied by Mallinckrodt Chemical Co. Ltd (USA). Commercial alumina discs (23 mm in diameter and 1.4 mm in thickness) were kindly supplied by Ghana Fine Ceramics (Taiwan) and used as a support substrate. 

### 2.2. Fabrication of the Hybrid PDMS Membranes by Water Emulsion 

The hybrid PDMS membranes were prepared using the water emulsion method as a green process [21]. A series of three hybrid sols were prepared using, as the starting materials, 4 g of Hy-PDMS and TEOS with 9.4, 12.6 and 15.7 wt.% (P9, P12, and P15), respectively. PDMS-OH/TEOS was mixed in solvent (H_2_O with 3 wt.% DBSA) with stirring of 600 rpm at 30 °C to obtain homogeneous solutions. After that, the catalyst (1.4 wt.% DBTDL) was added, and the hydrolysis–condensation reaction between Hy-PDMS and TEOS proceeded. The coating solutions obtained the appropriate viscosity during the specific reaction time, which were coated on alumina discs twice in a spin coater and each time for 30 s. Finally, the prepared membranes were placed into an oven immediately to cure at a specified temperature (75 or 120 °C) for 12 h. The test surface area of the prepared membranes was 13.7 cm^2^. 

### 2.3. Characterizations

The viscosities (*η*) of the coating solutions were measured by a controlled-strain viscometer (HBDV-ll+P, Brookfield engineering laboratories, Middleboro, MA, USA) at 25 °C. The Fourier transform infrared (FTIR) spectra of the membranes were characterized using a Jasco 4100 spectrophotometer equipped with an attenuated total reflectance accessory. Thermogravimetric (TGA) curves were determined by a thermal analyzer (TGA/DSC, Perkin Elmer, STA 6000) ranging from 50 to 600 °C with a heating rate of 10 °C/min in a nitrogen atmosphere. The surface morphologies of the hybrid PDMS membranes were obtained using atomic force microscopy (AFM; BRUKER Dimension Icon). The surface roughness of membranes was acquired using AFM images (scan field: 5 × 5 μm), recorded in tapping mode. The thickness of the membranes was measured from the cross-section morphologies obtained using scanning electron microscopy (SEM; JEOL JSM-6700F microscope) at an accelerating voltage of 3.0 kV. 

### 2.4. Membrane Performance Evaluation

The pure gas permeance of the prepared the hybrid PDMS membranes for H_2_, CO_2_, O_2_, N_2_, and CH_4_ were measured at a constant temperature (298 K) using a variable pressure/constant volume gas-permeation apparatus. The feed-side pressure of the gases ranged from 1 to 5 atm. Performance tests were conducted on at least three different samples to obtain an average. In Equation (1), the gas permeance (*P_GPU_*) of the membranes was estimated by the pressure increase on the downstream side of the cell using a pressure transducer and digital equipment connected to a computer (1 GPU = 7.5 × 10^−12^ m^3^ (STP)/m^2^ ·s·Pa).

(1) PGPU=273×1010760VATp×7614.7dpdt

where *V* is the volume of the downstream chamber (cm^3^), *A* is the effective area of the membranes (cm^2^), and *dp* is the transmembrane pressure (cmHg).

The ideal selectivity (*α**_i, j_***) between two different gases in a membrane can be calculated using the ratio of the permeabilities of the two gases (*i* and *j*) according to Equation (2):

(2) $$\alpha_{i,j}=\frac{P_{i}}{P_{j}}$$

## 3. Results and Discussion

### 3.1. Effect of TEOS on the Crosslinking Properties of PDMS in Water Emulsion

#### 3.1.1. Viscosity of the Coating Solution 

**Figure 1:**
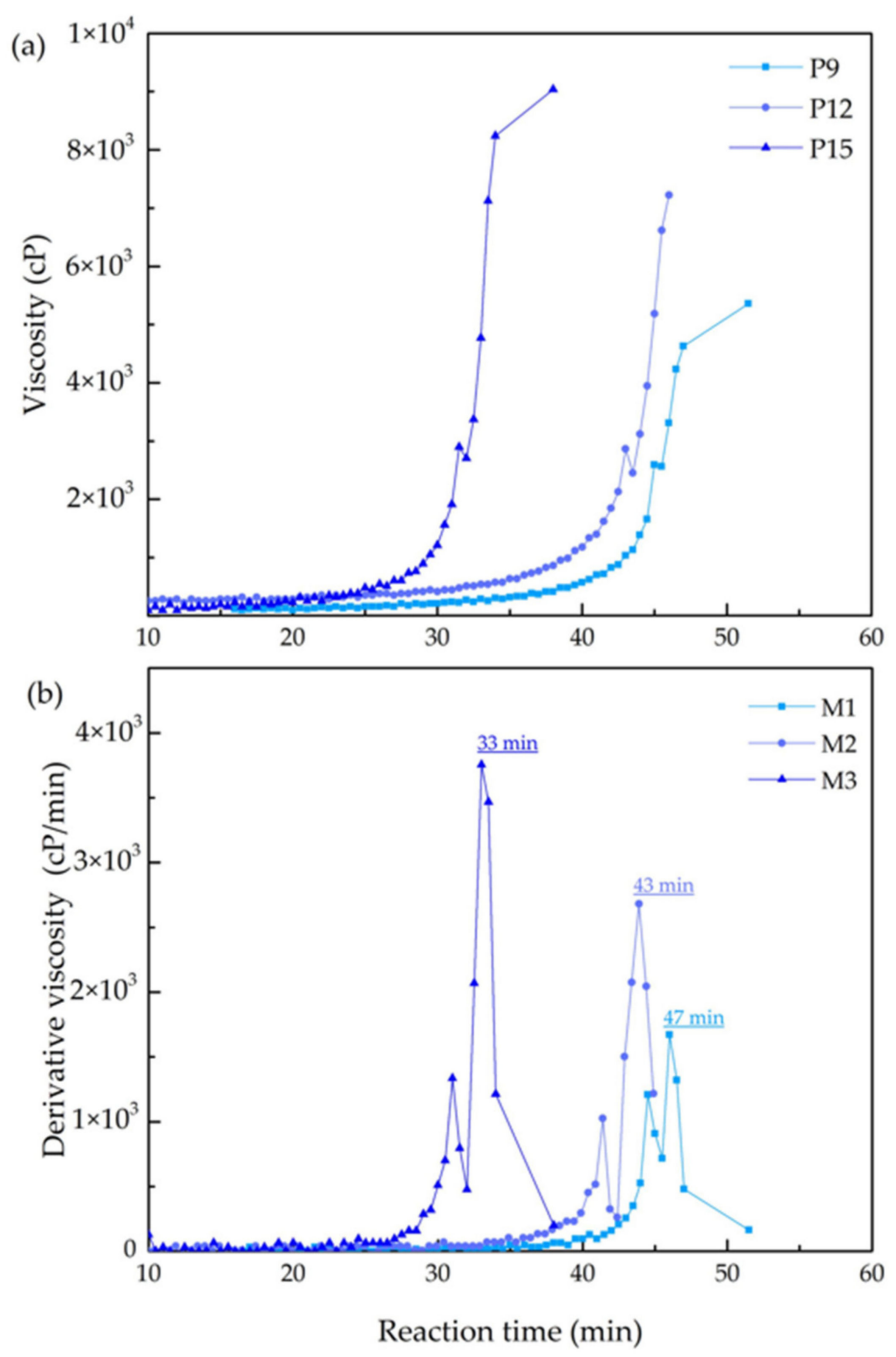
Time dependences of the (**a**) viscosity and (**b**) derivative viscosity of the coating solution in water emulsion during the crosslinking reaction with different TEOS loading ratios (9.4, 12.6, and 15.7 wt.%) at 27 °C.

Figure 1a shows the viscosity measurements of the PDMS solutions in water emulsion containing 9.4, 12.6 and 15.7 wt.% of TEOS were performed at 27 °C. It was found that the enhancement of viscosity with the time of crosslinking (as the reaction time) increased as the TEOS concentration increased. As shown in Figure 1b, the viscosity of the solution experienced a significant viscosity change in a specific reaction time, and the PDMS solutions were in the order of 15.7 wt.% (33 min) < 12.6 wt.% (43 min) < 9.4 wt.% (47 min). A significant change in the degree of crosslinking occurred. The obtained results are consistent with the literature [34] showing that the TEOS concentration affects the crosslinking rate of PDMS. 

#### 3.1.2. The Crosslinking Properties of the hybrid PDMS membranes 

**Figure 2:**
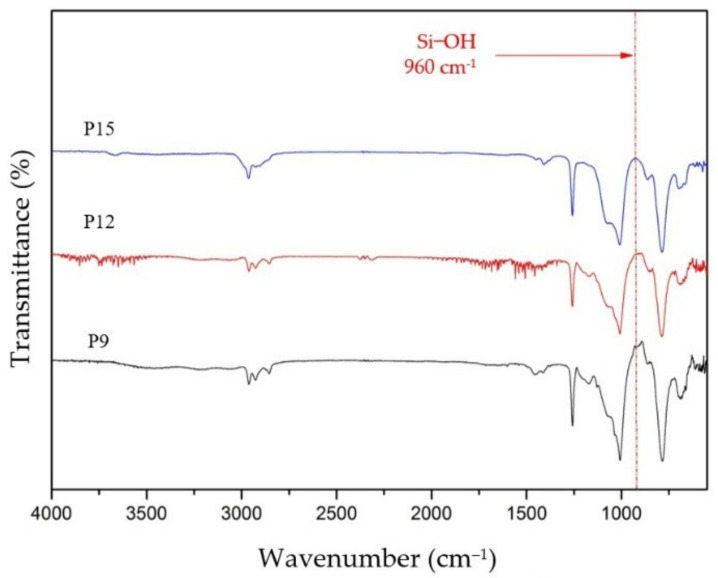
Fourier transform infrared (FTIR) spectra of the hybrid PDMS membranes with different TEOS loading ratios (9.4, 12.6 and 15.7 wt.%).

The crosslinking properties of the hybrid PDMS membranes were confirmed by FTIR spectra (Figure 2). In order to avoid a thickness effect on the results, the membranes were prepared by coating the PDMS solutions containing different TEOS loading ratios on the Al_2_O_3_ substrate at a specific reaction time according the viscosity results. The hybrid PDMS membranes displayed a characteristic C–H peak at ~2950 cm^−1^ (asymmetric CH_3_ stretching) and 1260–1259 cm^−1^ (CH_3_ deformation) in Si–CH_3_ [35,36]. A peak at ~960 cm^−1^ was observed for Hy-PDMS, attributable to Si–OH [37,38]. Notably, the FTIR band assigned to the asymmetric CH_3_ stretching (2950–2960 cm^−1^) enhanced in intensity alongside the increase in the TEOS concentration in the coating solution. However, the intensity of Si–OH (~960 cm^−1^) showed the opposite trend as the TEOS concentration increased. The results could be correlated with the crosslinking properties at the different PDMS/TEOS ratios [39]. The absence of a peak at ~960 cm^−1^ in P15 is confirmation of the completion of condensation reactions between the Hy-PDMS surface (silanol groups –OH) and TEOS. However, the high condensation rate could result in the existence of CH_3_ in the hybrid PDMS membranes due to the existence of incomplete hydrolysis of TEOS. By contrast, a low TEOS concentration in the solution is not enough to react completely with the all –OH group of Hy-PDMS, resulting in the existence of OH groups in the membranes (P9 and P12).

### 3.2. Effect of the Reaction Time on the Characterization of the Hybrid PDMS Membranes

#### 3.2.1. Morphological Characterization 

**Figure 3:**
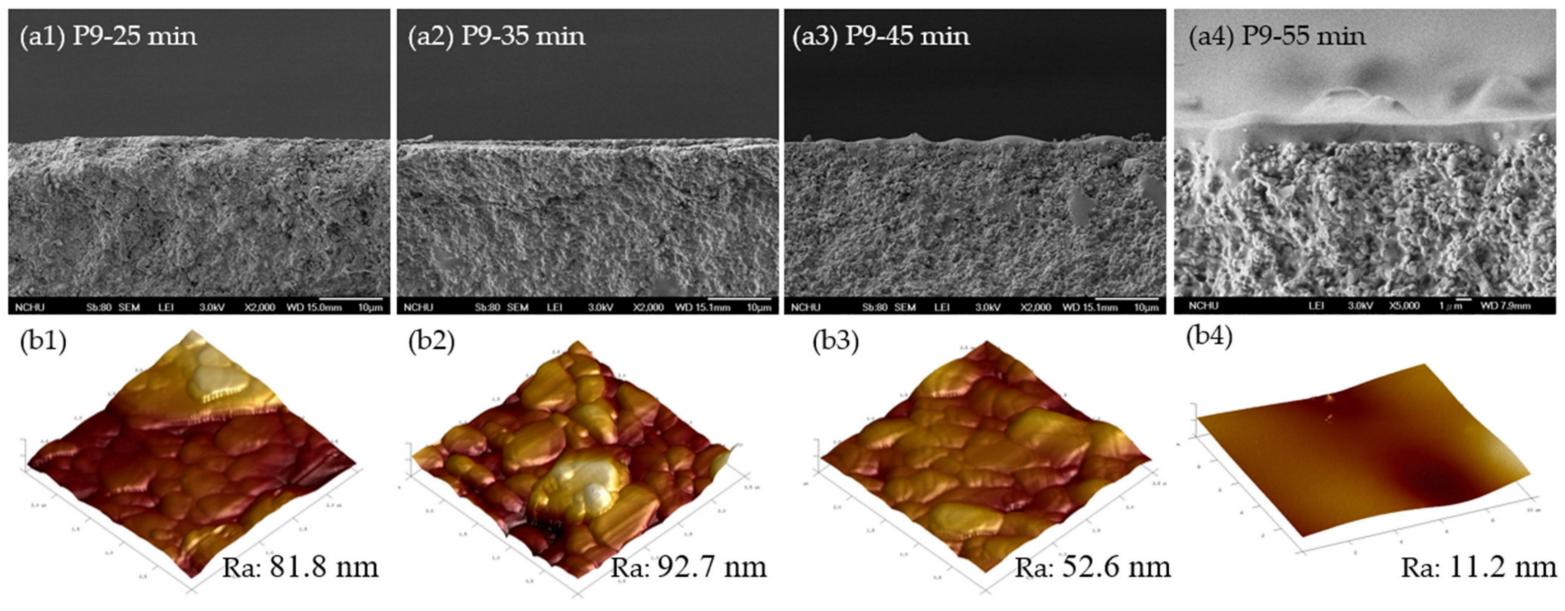
Scanning electron microscopy (SEM) (**a**) and atomic force microscopy (AFM) (**b**) images of the P9 membrane prepared at different reaction times: (**a1**,**b1**) 25 min, (**a2**,**b2**) 35 min, (**a3**,**b3**) 45 min and (**a4**,**b4**) 55 min. Ra, surface roughness.

**Figure 4:**
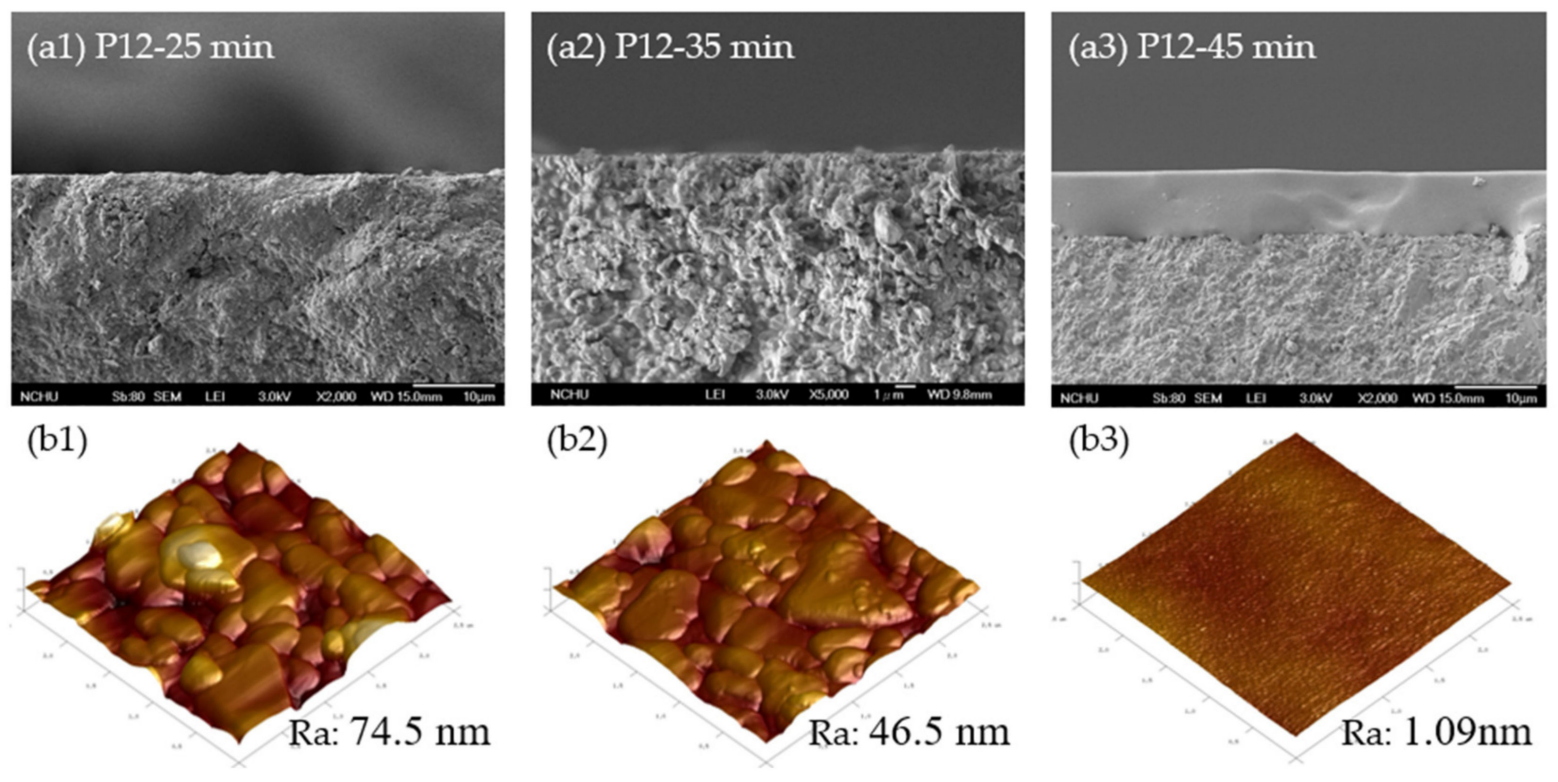
SEM (**a**) and AFM (**b**) images of the P12 membrane prepared at different reaction times: (**a1**,**b1**) 25 min, (**a2**,**b2**) 35 min and (**a3**,**b3**) 45 min.

**Figure 5:**
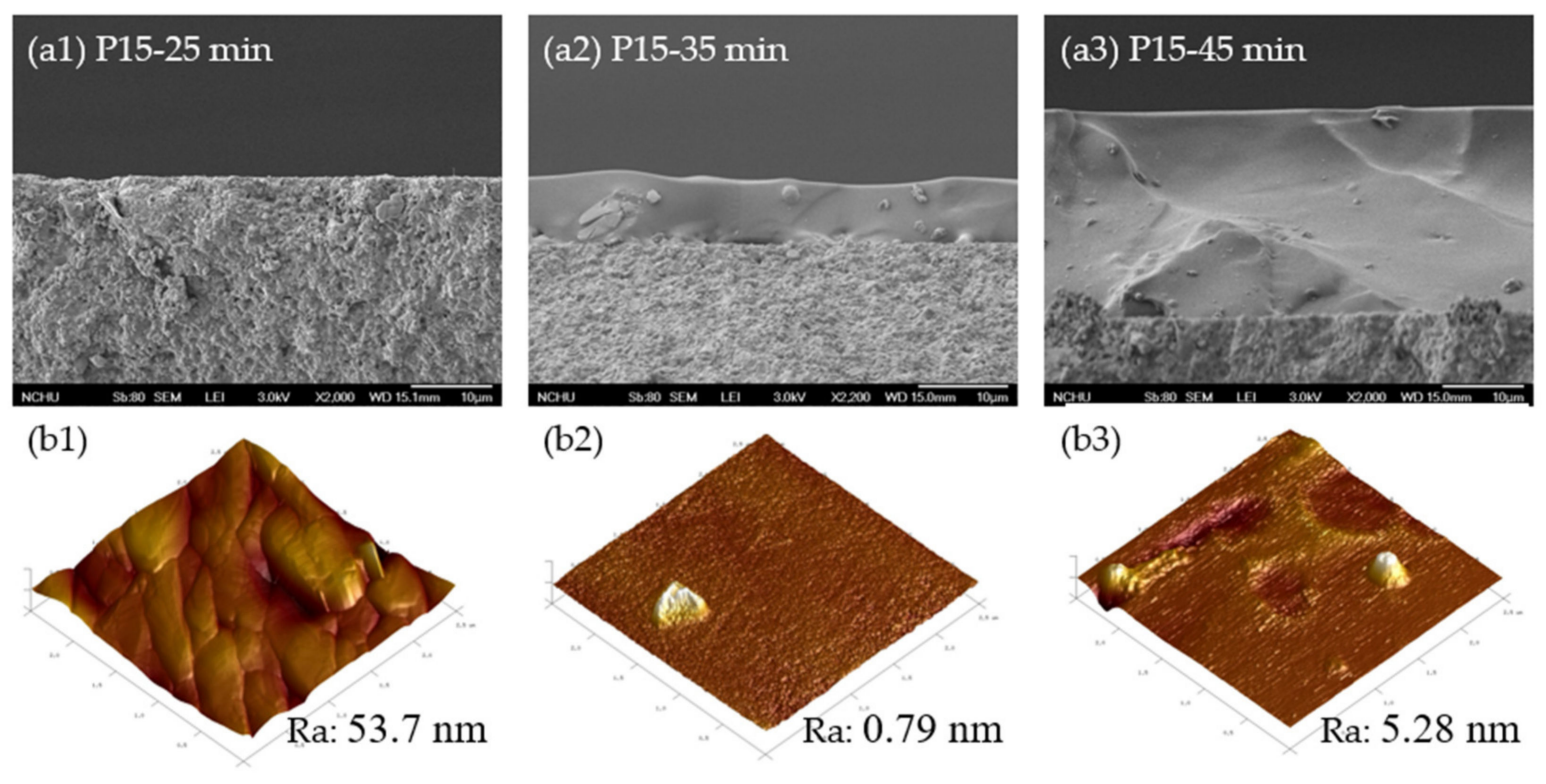
SEM (**a**) and AFM (**b**) images of the P15 membrane prepared at different reaction times (**a1**,**b1**) 25 min, (**a2**,**b2**) 35 min and (**a3**,**b3**) 45 min.

Varying the viscosity of the PDMS solutions during different reaction times can impart significant effects on the morphology of the membranes [40]. The cross-section and surface morphologies of the prepared membranes at different reaction times (25, 35, 45 and 55 min) were performed by SEM and AFM, as shown in Figure 3, Figure 4 and Figure 5. Increasing the reaction time from 25 to 45 min in the P9 membrane did not reveal a significant PDMS layer on the Al_2_O_3_ substrate (Figure 3(a1–a3)), but did show a change in the surface roughness (Ra) of 52~92 nm (Figure 3(b1–b3)). Even the long reaction time at 55 min in the P9 membrane resulted in a thin PDMS layer (~1 μm) on the substrate surface (Figure 3(a4)), as well as a decrease in surface roughness to 11.2 nm (Figure 3(b4)). However, P12 and P15 obtained asymmetric hybrid PDMS membranes when the reaction time was higher than 35 and 25 min, respectively (Figure 4 and Figure 5). P12 at 45 min had a PDMS layer thickness of 7.46 μm and a low surface roughness of 1.09 nm (Figure 4(a3,b3)). P15 at 35 min and P15 at 45 min had PDMS layer thicknesses/surface roughnesses of 7.47 μm/0.79 nm and 25.28 μm/5.28 nm, respectively (Figure 4(a2,a3,b2,b3)). The results could be correlated with the viscosity properties of the coating solution [41]. Before a significant crosslinking occurs in a PDMS solution, the low viscosity of the solution will most likely penetrate the substrate surface during the coating process. On the contrary, a high viscosity of the solution will form a dense PDMS layer on the substrate surface when a significant crosslinking occurs in the PDMS solution [42]. 

#### 3.2.2. Gas Permeation Properties 

**Figure 6:**
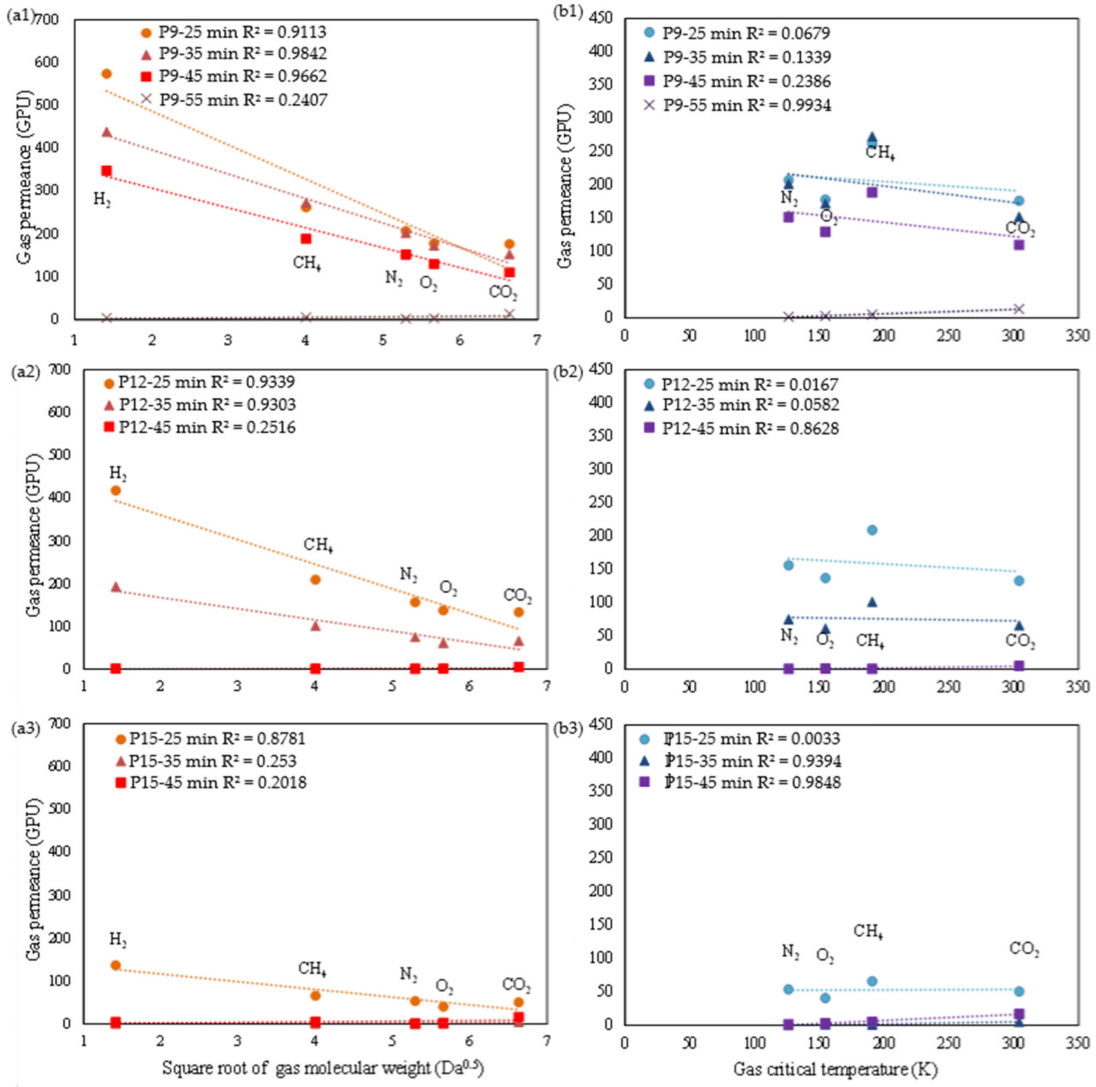
Correlation analysis between single gas permeance and (**a**) the square root of the gas molecular weight and (**b**) the gas critical temperature for the hybrid PDMS membranes: (**a1**,**b1**) P9, (**a2**,**b2**) P12, and (**a3**,**b3**) P15.

Figure 6 presents the gas permeance of the different gas molecules (H_2_, CO_2_, N_2_, O_2_, and CH_4_) in the hybrid PDMS membranes as a function of the square root of the molecular weights and critical temperatures, respectively. For the P9 membrane, the permeance follows the order of the square root of the molecular weights: H_2_ > CH_4_ > N_2_ > O_2_ > CO_2_, indicative of the strong Knudsen diffusion [43] (Figure 6(a1)). A linear dependence was observed with the linear coefficient of determination (R^2^) values of 0.91, 0.98, and 0.97 for the reaction times of 25, 35, and 45 min, respectively. When the reaction time increased to 55 min, the P9 membrane did not show a linear dependence, with a decrease in the coefficient to 0.24. The P12 membrane also had the same linear R^2^ = 0.93 for a reaction time of 25–35 min (Figure 6(a2)), but that showed a decrease in the coefficient to 0.25 when the reaction time increased to 45 min. A similar trend was seen for the P15 membrane, with decreases in coefficient from 0.88 to 0.20 when the reaction time increased from 25 min to 45 min (Figure 6(a3)). 

However, the opposite change trend was observed in Figure 6(b1–b3). P12 at 25 min, P15 at 35 min and P15 at 45 min showed a positive linear regression correlation between the permeance and critical temperature, indicative of strong solution diffusion [44], with R^2^ = 0.86, 0.94 and 0.98, respectively. Generally, the gas transport mechanism is relative to the morphological characterization. Knudsen diffusion and solution diffusion can occur in porous and dense membrane structures, respectively [45,46].

Table 1 shows the differences between the reaction times for the membrane preparation and the specific reaction times of the viscosity analysis, and that compared to the gas permeation properties of the membranes. Knudsen diffusion was the main gas transport mechanism for the hybrid PDMS membranes prepared below a specific reaction time. On the contrary, the solution diffusion mechanism was the main gas transport mechanism for the hybrid PDMS membranes prepared above a specific reaction time. According to the morphology of the prepared hybrid PDMS membranes, the structure changes from a porous symmetric membrane to an asymmetric membrane with a dense selective layer when the increase in the viscosity of the coating solution increases alongside the reaction time. Thus, the gas permeation property changes from Knudsen diffusion to solution diffusion as the pore size deceases. In other words, the asymmetric hybrid PDMS membranes can be prepared by the coating solution above a specific reaction time, which is suitable for application to CO_2_ separation by the solution diffusion mechanism. 

**Figure 7:**
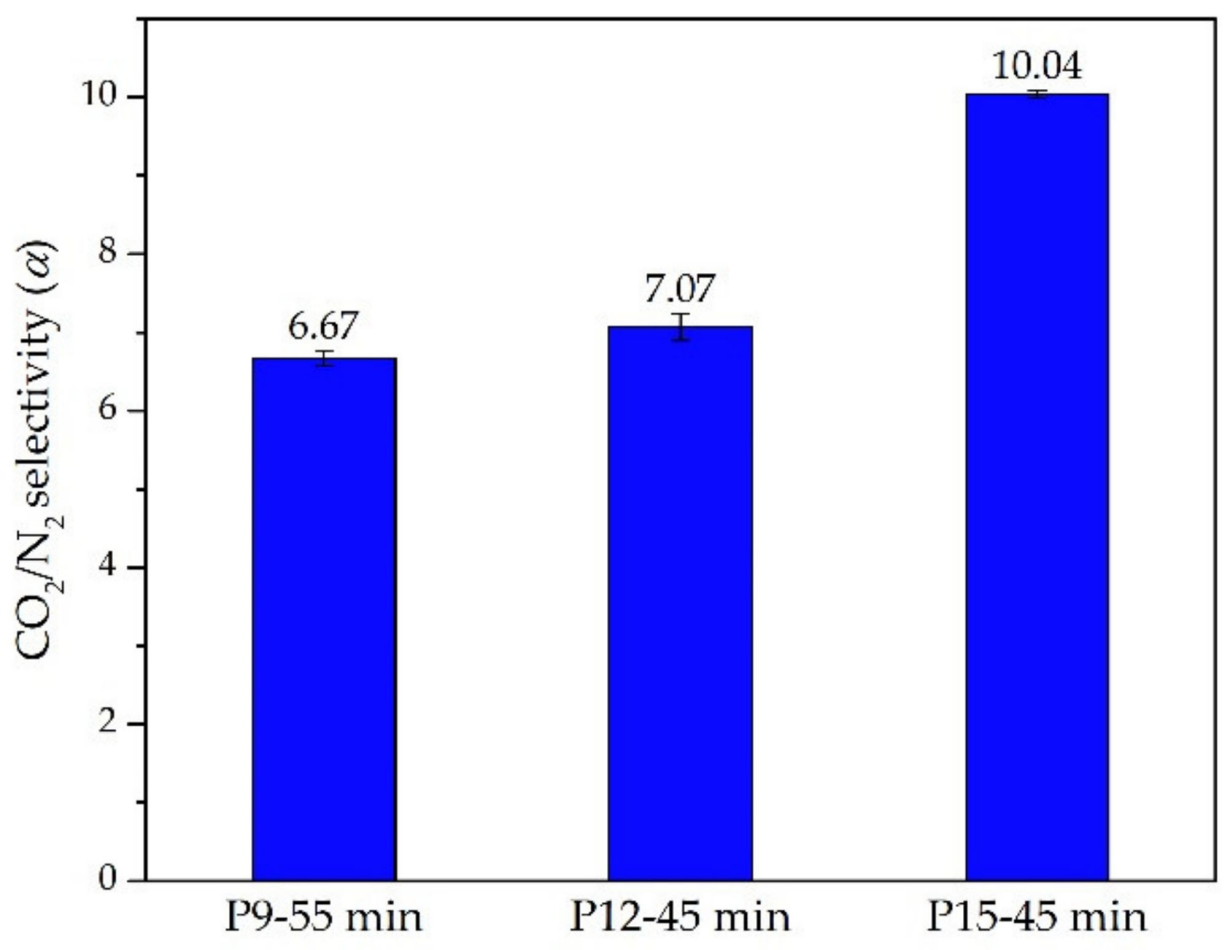
The CO_2_/N_2_ selectivity for P9-55 min, P12-45 min and P15-55 min at 25 °C.

On the other hand, the influence of the TEOS concentration on the CO_2_/N_2_ selectivity of the asymmetric hybrid PDMS membranes (P9-55 min, P12-45 min and P15-55 min) was investigated at 25 °C (Figure 7). When the TEOS concentration was increased, CO_2_/N_2_ selectivity increased from 6.67 (9.4 wt.%) to 10.04 (15.7 wt.%). This higher CO_2_/N_2_ selectivity was due to the crosslinking properties of hybrid PDMS membranes, which are considered in the FTIR results (Section 3.1.2). The P15 membrane, with its high TEOS concentration, shows a fully crosslinked structure when compared to the P9 and P12. Moreover, the crosslinking structure of the PDMS membrane can induce high solution diffusion for CO_2_/N_2_ separation [47]. Therefore, higher selectivity was observed for the P15 membranes, owing to their crosslinked structure.

### 3.3. CO_2_/N_2_ Sepaparation of the Hybrid PDMS Membrane

**Figure 8:**
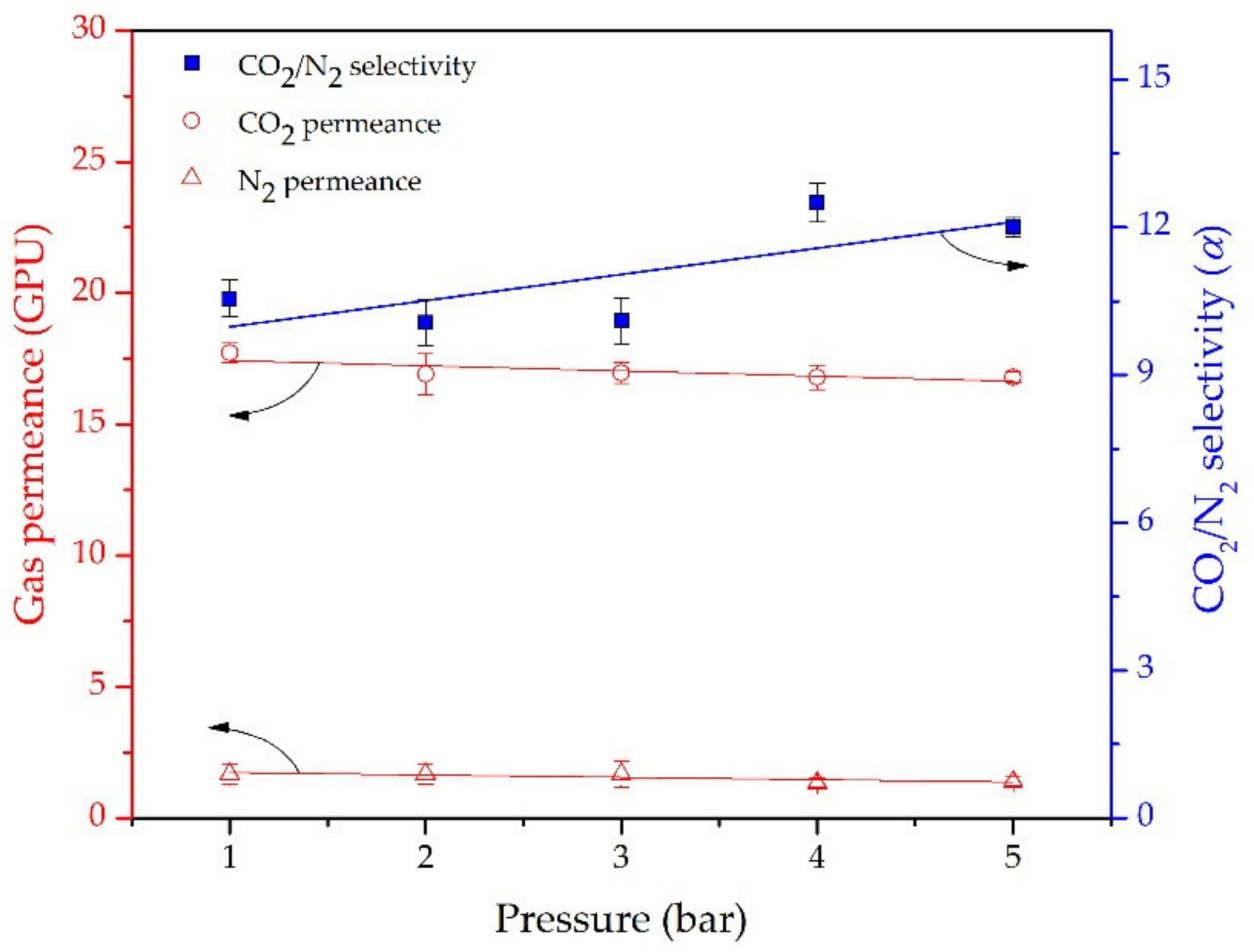
Effect of feed pressure on the gas permeance and ideal selectivity of CO_2_/N_2_ at 25 °C for the P15 membrane.

Figure 8 indicates the effect of feed pressure on CO_2_ and N_2_ permeance and selectivity at 25 °C by varying the feed pressure from 1 to 5 bar for single gases. It presents a slight drop in permeance with increasing feed pressure, especially in N_2_ permeance, which decreases with an increase in feed pressure from 3 to 5 bar. Thus, it is obvious that the CO_2_/N_2_ selectivity increases as the feed pressure increases. 

According to the solution diffusion properties of penetrants, condensable gases (CO_2_ or hydrocarbon) exhibit an enhancement in solubility as the feed pressure increases in rubbery polymers. The gas concentration in the polymer matrix increases as the pressure increases, which could further result in the plasticization of the polymer matrix over a critical pressure. The plasticized polymer chains in the membrane would show high gas permeance due to the chain flexibility. In contrast, the hydrostatic compression as the dominant factor for inert gas (H_2_, O_2_, N_2_, etc.) could result in free volume in the matrix and, hence, a decrease in permeance as the feed pressure increases. Herein, the gas permeance was mainly decreased by the compression effect due to a decrease in the free volume. Moreover, the reduction in N_2_ permeance was more than that in CO_2_ permeance at a high feed pressure, so the CO_2_/N_2_ selectivity increased. 

However, it is important to note that the ideal selectivity can increases as the pressure increases for a single gas, but the real selectivity could decrease for mixed gases by means of the coupling effect [3]. In other words, the P15 membrane could have a weak stability in the polymer structure, which should be improved by further modification.

### 3.4. Enhancing CO_2_ Permeance Using the Curing Process

**Figure 9:**
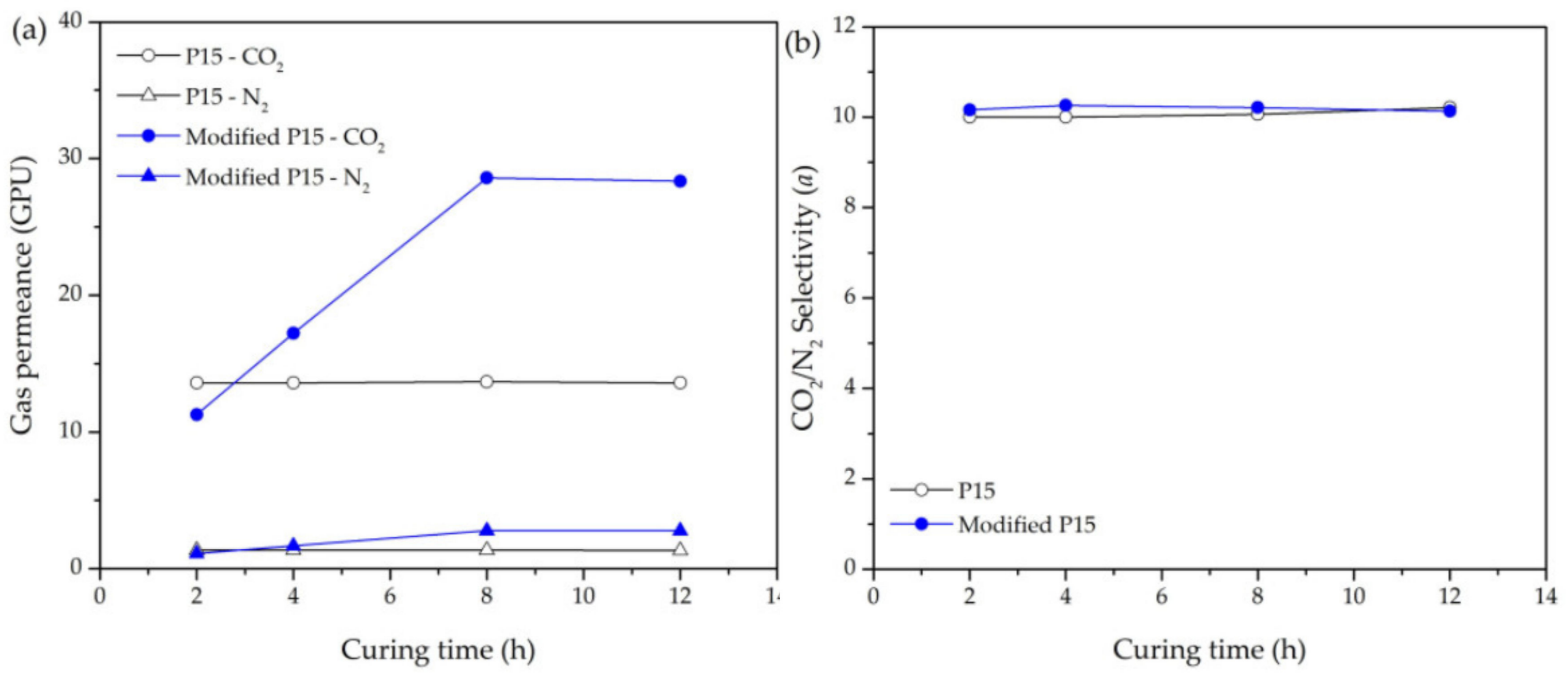
(**a**) The gas permeance and (**b**) ideal selectivity for CO_2_/N_2_ at 25 °C for P15 and the modified P15 membrane as a function of curing time.

The P15 membrane was modified by increasing the curing temperature from 75 to 120 °C. Figure 9 shows the CO_2_/N_2_ separation for P15 and the modified P15 membrane with respect to changes in curing time ranging from 2 to 12 h. As shown in Figure 9a, both gases permeabilities of the P15 membrane remained relatively unchanged under the different curing time. However, for the modified P15 membrane, it was observed that both permeabilities increased as the curing time increased to 8 h, and then remained almost constant with curing times of 8–12 h. 

**Figure 10:**
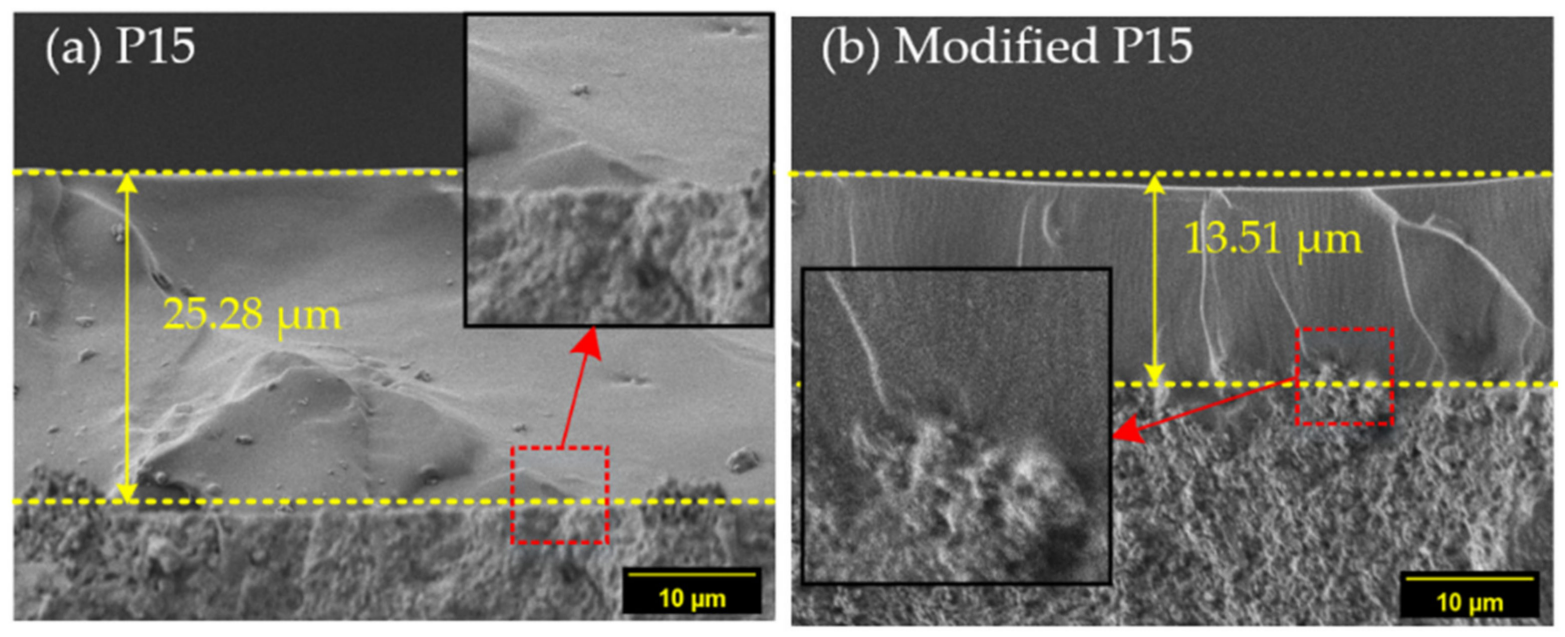
SEM images of the PDMS layer supported on the ceramic substrate: (**a**) P15 and (**b**) modified P15 prepared at 75 and 120 °C for 12 h, respectively.

The thickness of the PDMS layer on the support is one of the dominant factors affecting gas permeation performance [48,49]. The thickness of the PDMS layers, measured using SEM images for two membranes, are compared in Figure 10. The SEM images show that the thickness of the PDMS layer of the P15 membrane was 25.28 μm (Figure 10a). Compared to the P15 membrane, the modified P15 membrane showed a 53% reduction in thickness to 13.51 μm (Figure 10b). Moreover, it is interesting to note that the modified P15 membrane showed greater interfacial adhesion compared to the P15 membrane. In general, the curing temperature can increase the liquidity of polymer solutions due to a decrease in viscosity [50,51]. In other words, a thin PDMS layer was formed on the ceramic substrate by enhancing the permeability of the coating solution on the surface of the support, which increased the gas permeance. 

**Figure 11:**
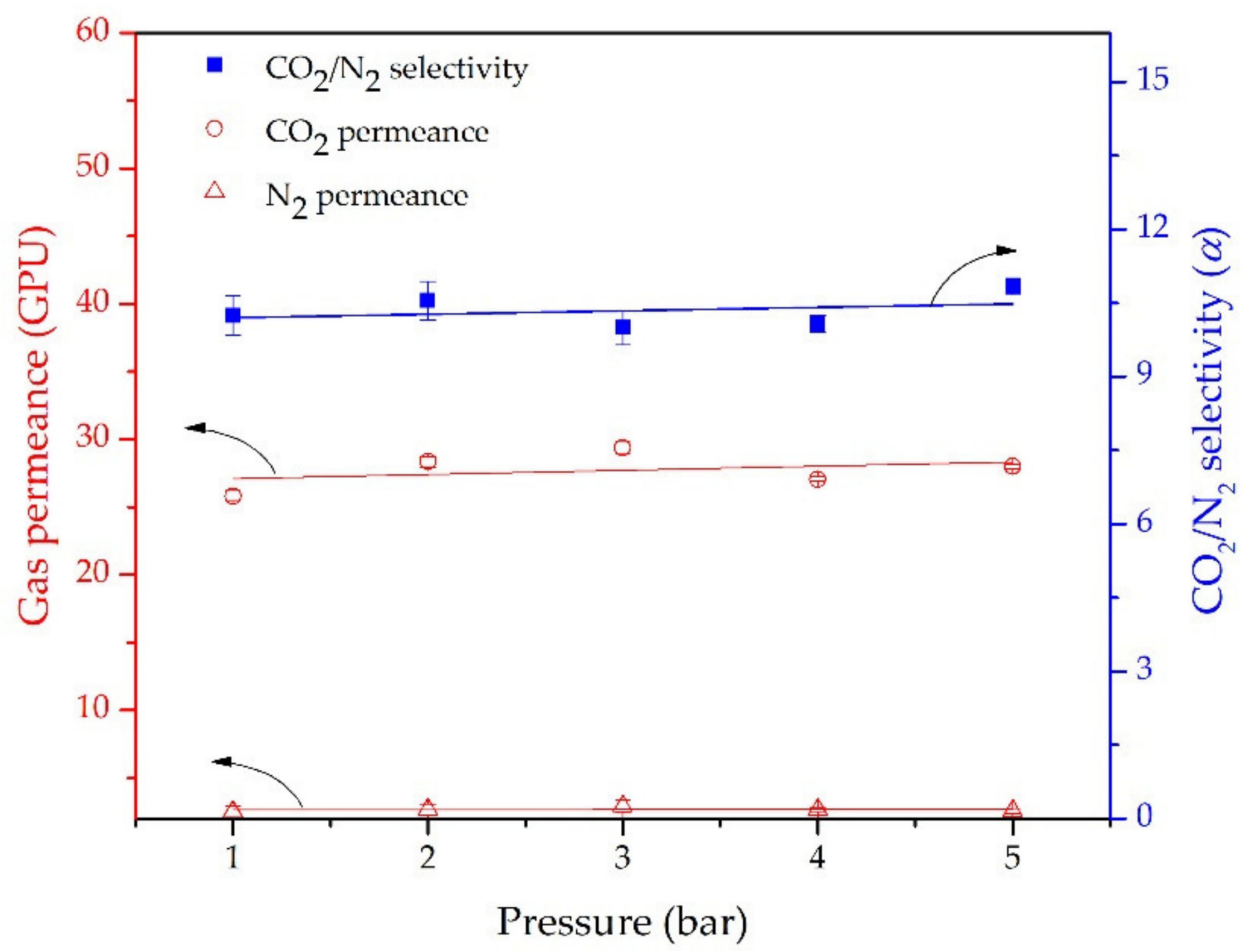
Effect of feed pressure on the gas permeance and ideal selectivity of CO_2_/N_2_ at 25 °C for the modified P15 membrane.

**Figure 12:**
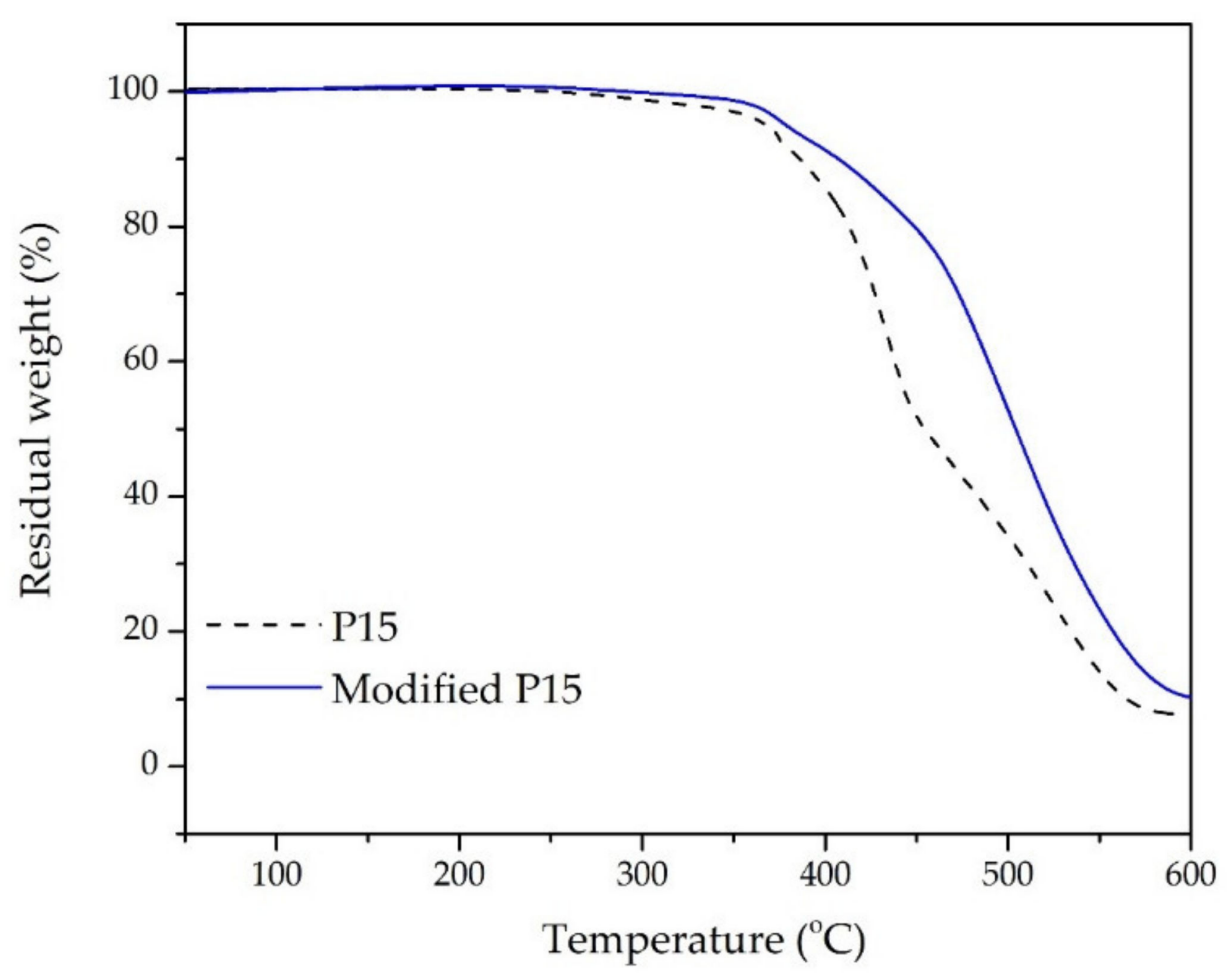
Thermogravimetric (TGA) curves of the P15 and the modified P15.

Figure 9b shows the CO_2_/N_2_ selectivity for the two membranes, which stayed at an almost constant value as the curing time increased. Thus, the change in thickness only affected the gas permeance, not the selectivity. The CO_2_/N_2_ separation performance of the modified P15 membrane measured at different feed pressures is provided in Figure 11. It was clearly observed that gas permeance is independent of the feed pressure, as the modified P15 membrane exhibited stable CO_2_/N_2_ selectivity as the pressure increased. As shown in Figure 12, the thermal properties of the P15 and the modified P15 were further measured by TGA for evaluating the stability of the crosslinked structure. The decomposition temperature (*T_d_*) of P15 membranes shift towards a higher temperature after modification. The 10 wt% decomposition temperature (*T_d_*_10_) increased from 386 °C (P15) to 403 °C (modified P15). The results indicate that the modified P15 shows a high stability due to its crosslinked structure when compared to the P15. Therefore, it was concluded that the high stability of the crosslinked structure could provide a stable gas separation performance at different operating pressures. 

Table 2 shows a comparison between this work and other studies to evaluate the CO_2_/N_2_ separation performance of the hybrid PDMS membranes. For the most part, the developed hybrid PDMS membranes prepared by a green solvent exhibited better CO_2_/N_2_ selectivity compared to the membranes prepared by an organic solvent. However, the developed membranes had relatively low gas permeance compared to others. The low molecular weight of the PDMS polymer could form a dense molecular structure in the membranes, resulting in the low gas transport property. Thus, there are several suggestions for obtaining greater performance. First, an alternative fabrication method could be to create high-performance hybrid PDMS membranes with a highly effective permeation area and a thin selective layer, for example, the development of thin-film tubular or hollow fiber composite membranes using a novel coating technique [52,53,54]. Second, using a PDMS polymer with high molecular weight could further enhance the gas permeance by increasing the porosity or pore size [55,56]. Last, the choice of crosslinker can more accurately control the diffusion channel construction of hybrid PDMS membranes, such as organosilica with different molecular sizes [57].

## 4. Conclusions

A process for preparing asymmetric hybrid PDMS membranes using the green sol–gel method in water emulsion was described herein. The effects of several parameters, including the crosslinker (TEOS) concentration and curing temperature, on the CO_2_/N_2_ separation performance of membranes were successfully investigated. The hybrid PDMS, containing 15 wt.% TEOS, displayed a shorter specific reaction time (~33 min) for forming fully crosslinked structures on the substrate, as demonstrated by the viscosity analysis and FTIR spectroscopy. Thus, the hybrid PDMS membrane, prepared at reaction time of 45 min (P15-45 min), showed the highest CO_2_/N_2_ selectivity at 10.04. Furthermore, it was found that a high curing temperature of 120 °C resulted in a membrane with a high gas permeation performance. This was ascribed to the decrease in the thickness of the hybrid PDMS membrane from 25.28 μm to 13.51 μm, as well as the high stability of the crosslinked structure. This was demonstrated by SEM and TGA measurement. This thin state resulted in an increased CO_2_ permeance to from 13.6 GPU to 28.3 GP. Moreover, the developed membrane showed good resistance to compression effects due to its stable crosslinked structure, leading to constant CO_2_/N_2_ selectivity under different feed pressures. The outcomes of this study show that we have developed a potential green approach to synthesize hybrid PDMS membranes with high CO_2_ separation performance, which can be readily applied in various industrial, cement and petrochemical industries for separating CO_2_ from other gases.

## Figures and Tables

**Table 1:** Reaction times of the membrane preparation and the correlation of permeance with molecular weight and critical temperature for the hybrid PDMS membranes.

Membrane	Specific Reaction Time by Viscosity (min)	Time Difference (∆*t*, min) ^1^	Correlation between Gas Permeance and Gas Properties (*R*^2^)	
			Molecular Weight	Critical Temperature
P9-25 min	47	−22	0.91	0.06
P9-35 min		−12	0.98	0.13
P9-45 min		−2	0.97	0.23
P9-55 min		+8	0.24	0.99
P12-25 min	43	−18	0.93	0.52
P12-35 min		−8	0.93	0.17
P12-45 min		+2	0.25	0.86
P15-25 min	33	−8	0.88	0.01
P15-35 min		+2	0.25	0.94
P15-45 min		+12	0.20	0.98

^1^ Time difference (∆*t*) = [the prepared reaction time (coating time)] − (the specific reaction time by viscosity).

**Table 2:** CO_2_ and N_2_ permeances and CO_2_/N_2_ selectivity of the PDMS membranes measured in this work compared to those reported in the literature.

Membrane	Substrate ^1^	Solvent	Permeance (GPU)		CO_2_/N_2_ (α)	M.W. of PDMS (Da) ^2^	Ref.
			CO_2_	N_2_			
PDMS/~100 μm	-	toluene	47.01 ± 2	6.3 ± 0.2	7.5	-	[7]
PDMS/25 μm	HMS	n-heptane	108.76 ± 2	11.5 ± 0.3	9.5	-	[5]
PDMS/14 μm	CA	n-heptane	117.9	14.4	8.2	5000	[6]
PERVAP 4060/~10 μm	HMS	-	475.7 ± 63	56.4 ± 20	8.4	-	[58]
PDMS/8–9 μm	ZrO_2_/Al_2_O_3_	n-heptane	410.9	47.8	8.6	60,000	[59]
PDMS/6 μm	TiO_2_/PES	n-hexane	80~95	-	5.5~6.3		[4]
P15/13.5 μm	Al_2_O_3_ disc	water	27.7 ± 1.3	2.7 ± 0.2	10.3 ± 0.3	4200	This work

^1^ HMS, highly microporous support; CA, cellulose acetate; TiO_2_/PES was prepared by coating TiO_2_ onto the polyethersulfone (PES) support. ^2^ M.W., Molecular weight.

## Data Availability

Not applicable.

## Nomenclature

*P_GPU_*	Gas permeance (GPU, 7.5 × 10^−12^ m^3^ (STP)/m^2^·s·Pa)
*V*	Volume of the downstream chamber (cm^3^)
*A*	Effective area of the membranes (cm^2^)
*T*	Temperature (K)
*dp*	Transmembrane pressure (cmHg)
*dt*	Time (s)
*α_i, j_*	Ideal selectivity of two gases *i* and *j*
Ra	Surface roughness (nm)

## Abbreviations

AFM	Atomic Force Microscopy
CA	Cellulose Acetate
DBSA	4-Dodecylbenzenesulfonic acid
DBTDL	Dibutyltin Dilaurate
FTIR	Fourier Transform Infrared
GPU	Gas Permeation Unit
HMS	Highly Microporous Support
Hy-PDMS	Hydroxyl-terminated Polydimethylsiloxane
M.W.	Molecular weight
PDMS	Polydimethylsiloxane
PES	Polyethersulfone
SDS	Sodium Dodecyl sulfate
SEM	Scanning Electron Microscopy
TEOS	Tetraethylorthosilicate
TGA	Thermogravimetric
TiO_2_	Titanium Dioxide

## References

[1] Norahim N., Yaisanga P., Faungnawakij K., Charinpanitkul T., Klaysom C. (2018). Recent Membrane Developments for CO_2_ Separation and Capture. Chem. Eng. Technol..

[2] Brandrup J., Immergut E.H., Grulke E.A. (1999). Polymer Handbook.

[3] Yeom C.K., Lee S.H., Lee J.M. (2000). Study of transport of pure and mixed CO_2_/N_2_ gases through polymeric membranes. J. Appl. Polym. Sci..

[4] Madaeni S.S., Badieh M.M.S., Vatanpour V., Ghaemi N. (2012). Effect of titanium dioxide nanoparticles on polydimethylsiloxane/polyethersulfone composite membranes for gas separation. Polym. Eng. Sci..

[5] Merkel T.C., Bondar V.I., Nagai K., Freeman B.D., Pinnau I. (2000). Gas sorption, diffusion, and permeation in poly(dimethylsiloxane). J. Polym. Sci. Part B Polym. Phys..

[6] Wu F., Li L., Xu Z., Tan S., Zhang Z. (2006). Transport study of pure and mixed gases through PDMS membrane. Chem. Eng. J..

[7] Sadrzadeh M., Shahidi K., Mohammadi T. (2010). Synthesis and gas permeation properties of a single layer PDMS membrane. J. Appl. Polym. Sci..

[8] Orooji Y., Movahedi A., Liu Z., Asadnia M., Ghasali E., Ganjkhanlou Y., Razmjou A., Karimi-Maleh H., Kiadeh N.T.H. (2020). Luminescent film: Biofouling investigation of tetraphenylethylene blended polyethersulfone ultrafiltration membrane. Chemosphere.

[9] Orooji Y., Jaleh B., Homayouni F., Fakhri P., Kashfi M., Torkamany M.J., Yousefi A.A. (2020). Laser Ablation-Assisted Synthesis of Poly (Vinylidene Fluoride)/Au Nanocomposites: Crystalline Phase and Micromechanical Finite Element Analysis. Polymers.

[10] Roslan R.A., Lau W.J., Lai G.S., Zulhairun A.K., Yeong Y.F., Ismail A.F., Matsuura T. (2020). Impacts of Multilayer Hybrid Coating on PSF Hollow Fiber Membrane for Enhanced Gas Separation. Membranes.

[11] Yang Y., Han Y., Pang R., Ho W.S.W. (2020). Amine-Containing Membranes with Functionalized Multi-Walled Carbon Nanotubes for CO_2_/H_2_ Separation. Membranes.

[12] Monteiro B., Nabais A.R., Casimiro M.H., Martins A.P.S., Francisco R.O., Neves L.A., Pereira C.C.L. (2018). Impact on CO_2_/N_2_ and CO_2_/CH_4_ Separation Performance Using Cu-BTC with Supported Ionic Liquids-Based Mixed Matrix Membranes. Membranes.

[13] Orooji Y., Ghasali E., Emami N., Noorisafa F., Razmjou A. (2019). ANOVA Design for the Optimization of TiO_2_ Coating on Polyether Sulfone Membranes. Molecules.

[14] Ebrahimi F., Orooji Y., Razmjou A. (2020). Applying Membrane Distillation for the Recovery of Nitrate from Saline Water Using PVDF Membranes Modified as Superhydrophobic Membranes. Polymers.

[15] Öztürk A., Bayrakçeken Yurtcan A. (2020). Investigation of synergetic effect of PDMS polymer hydrophobicity and polystyrene-silica particles roughness in the content of microporous layer on water management in PEM fuel cell. Appl. Surf. Sci..

[16] He X., Wang T., Huang J., Chen J., Li J. (2020). Fabrication and characterization of superhydrophobic PDMS composite membranes for efficient ethanol recovery via pervaporation. Sep. Purif. Technol..

[17] Ataeivarjovi E., Tang Z., Chen J. (2018). Study on CO_2_ Desorption Behavior of a PDMS–SiO_2_ Hybrid Membrane Applied in a Novel CO_2_ Capture Process. Acs Appl. Mater. Interfaces.

[18] Rosli A., Ahmad A.L., Low S.C. (2020). Enhancing membrane hydrophobicity using silica end-capped with organosilicon for CO_2_ absorption in membrane contactor. Sep. Purif. Technol..

[19] Chiun-Jye Y., Wei-Jen H. Application of TEOS/PDMS ormosil in the fabrication of amperometric biosensor. Proceedings of the IEEE International Workshop on Biomedical Circuits and Systems.

[20] Rao H., Zhang Z., Song C., Qiao T., Xu S. (2011). Gas separation properties of siloxane/polydimethylsiloxane hybrid membrane containing fluorine. Sep. Purif. Technol..

[21] Li S., Qin F., Qin P., Karim M.N., Tan T. (2013). Preparation of PDMS membrane using water as solvent for pervaporation separation of butanol-water mixture. Green Chem..

[22] Bai Y., Dong L., Zhang C., Gu J., Sun Y., Zhang L., Chen H. (2013). ZIF-8 Filled Polydimethylsiloxane Membranes for Pervaporative Separation of n-Butanol from Aqueous Solution. Sep. Sci. Technol..

[23] Riesco R., Boyer L., Blosse S., Lefebvre P.M., Assemat P., Leichle T., Accardo A., Malaquin L. (2019). Water-in-PDMS Emulsion Templating of Highly Interconnected Porous Architectures for 3D Cell Culture. ACS Appl. Mater. Interfaces.

[24] Si Z., Shan H., Hu S., Cai D., Qin P. (2018). Recovery of ethanol via vapor phase by polydimethylsiloxane membrane with excellent performance. Chem. Eng. Res. Des..

[25] Jiesheng L., Ke L., Lian X., Xiang H. (2016). Synthesis of Polysiloxanes In Microemulsion Via ring opening of D4. Nanochem. Res..

[26] Deka B.J., Lee E.-J., Guo J., Kharraz J., An A.K. (2019). Electrospun Nanofiber Membranes Incorporating PDMS-Aerogel Superhydrophobic Coating with Enhanced Flux and Improved Antiwettability in Membrane Distillation. Environ. Sci. Technol..

[27] Pagliaro M., Ciriminna R., Palmisano G., Levy D., Zayat M. (2015). Sol-Gel for Environmentally Green Products. The Sol-Gel Handbook—Synthesis, Characterization and Application.

[28] Ge M., Cao C., Liang F., Liu R., Zhang Y., Zhang W., Zhu T., Yi B., Tang Y., Lai Y. (2020). A “PDMS-in-water” emulsion enables mechanochemically robust superhydrophobic surfaces with self-healing nature. Nanoscale Horiz..

[29] Zhang W., Su X., Shi B., Wang L., Wang Q., Li S. (2015). Influence of molecular weight of polydimethylsiloxane precursors and crosslinking content on degree of ethanol swelling of crosslinked networks. React. Funct. Polym..

[30] Lee J.Y., Lee J.S., Lee J.-H. (2020). High performance and thermally stable PDMS pervaporation membranes prepared using a phenyl-containing tri-functional crosslinker for n-butanol recovery. Sep. Purif. Technol..

[31] Xiangli F., Chen Y., Jin W., Xu N. (2007). Polydimethylsiloxane (PDMS)/Ceramic Composite Membrane with High Flux for Pervaporation of Ethanol−Water Mixtures. Ind. Eng. Chem. Res..

[32] Stafie N., Stamatialis D.F., Wessling M. (2005). Effect of PDMS Crosslinking degree on the permeation performance of PAN/PDMS composite nanofiltration membranes. Sep. Purif. Technol..

[33] Berean K., Ou J.Z., Nour M., Latham K., McSweeney C., Paull D., Halim A., Kentish S., Doherty C.M., Hill A.J. (2014). The effect of crosslinking temperature on the permeability of PDMS membranes: Evidence of extraordinary CO_2_ and CH_4_ gas permeation. Sep. Purif. Technol..

[34] Téllez L., Rubio J., Rubio F., Morales E., Oteo J.L. (2003). Synthesis of inorganic-organic hybrid materials from TEOS, TBT and PDMS. J. Mater. Sci..

[35] Johnson L.M., Gao L., Shields Iv C.W., Smith M., Efimenko K., Cushing K., Genzer J., López G.P. (2013). Elastomeric microparticles for acoustic mediated bioseparations. J. Nanobiotechnol..

[36] Shafqat S.S., Khan A.A., Zafar M.N., Alhaji M.H., Sanaullah K., Shafqat S.R., Murtaza S., Pang S.C. (2019). Development of amino-functionalized silica nanoparticles for efficient and rapid removal of COD from pre-treated palm oil effluent. J. Mater. Res. Technol..

[37] Groza A., Surmeian A. (2015). Characterization of the Oxides Present in a Polydimethylsiloxane Layer Obtained by Polymerisation of Its Liquid Precursor in Corona Discharge. J. Nanomater..

[38] Kuo A.C.M. (1999). Polymer Data Handbook.

[39] Rao H.-X., Liu F.-N., Zhang Z.-Y. (2007). Preparation and oxygen/nitrogen permeability of PDMS crosslinked membrane and PDMS/tetraethoxysilicone hybrid membrane. J. Membr. Sci..

[40] Dong Z., Liu G., Liu S., Liu Z., Jin W. (2014). High performance ceramic hollow fiber supported PDMS composite pervaporation membrane for bio-butanol recovery. J. Membr. Sci..

[41] Calleja A., Ricart S., Aklalouch M., Mestres N., Puig T., Obradors X. (2014). Thickness–concentration–viscosity relationships in spin-coated metalorganic ceria films containing polyvinylpyrrolidone. J. Sol Gel Sci. Technol..

[42] Li Z., Han W., Kozodaev D., Brokken-Zijp J.C.M., de With G., Thüne P.C. (2006). Surface properties of poly(dimethylsiloxane)-based inorganic/organic hybrid materials. Polymer.

[43] Javaid A. (2005). Membranes for solubility-based gas separation applications. Chem. Eng. J..

[44] Shao L., Low B.T., Chung T.-S., Greenberg A.R. (2009). Polymeric membranes for the hydrogen economy: Contemporary approaches and prospects for the future. J. Membr. Sci..

[45] Oyama S.T., Yamada M., Sugawara T., Takagaki A., Kikuchi R. (2011). Review on Mechanisms of Gas Permeation through Inorganic Membranes. J. Jpn. Pet. Inst..

[46] Ismail A.F., Khulbe K.C., Matsuura T. (2015). Fundamentals of Gas Permeation Through Membranes. Gas Separation Membranes: Polymeric and Inorganic.

[47] Kim D., Hossain I., Kim Y., Choi O., Kim T.-H. (2020). PEG/PPG-PDMS-Adamantane-based Crosslinked Terpolymer Using the ROMP Technique to Prepare a Highly Permeable and CO(2)-Selective Polymer Membrane. Polymers.

[48] Moradi M.R., Pourafshari Chenar M., Noie S.H. (2016). Using PDMS coated TFC-RO membranes for CO_2_/N_2_ gas separation: Experimental study, modeling and optimization. Polym. Test..

[49] Madaeni S.S., Badieh M.M.S., Vatanpour V. (2013). Effect of coating method on gas separation by PDMS/PES membrane. Polym. Eng. Sci..

[50] Brunchi C.-E., Bercea M., Morariu S., Dascalu M. (2016). Some properties of xanthan gum in aqueous solutions: Effect of temperature and pH. J. Polym. Res..

[51] Selyanchyn R., Ariyoshi M., Fujikawa S. (2018). Thickness Effect on CO_2_/N_2_ Separation in Double Layer Pebax-1657^®^/PDMS Membranes. Membranes.

[52] Dong Z., Zhu H., Hang Y., Liu G., Jin W. (2020). Polydimethylsiloxane (PDMS) Composite Membrane Fabricated on the Inner Surface of a Ceramic Hollow Fiber: From Single-Channel to Multi-Channel. Engineering.

[53] Khulbe K.C., Matsuura T. (2018). Thin film composite and/or thin film nanocomposite hollow fiber membrane for water treatment, pervaporation, and gas/vapor separation. Polymer.

[54] Chen H.Z., Thong Z., Li P., Chung T.-S. (2014). High performance composite hollow fiber membranes for CO_2_/H_2_ and CO_2_/N_2_ separation. Int. J. Hydrog. Energy.

[55] Gurr P.A., Scofield J.M.P., Kim J., Fu Q., Kentish S.E., Qiao G.G. (2014). Polyimide polydimethylsiloxane triblock copolymers for thin film composite gas separation membranes. J. Polym. Sci. Part A Polym. Chem..

[56] Cao P.-F., Li B., Hong T., Xing K., Voylov D.N., Cheng S., Yin P., Kisliuk A., Mahurin S.M., Sokolov A.P. (2017). Robust and Elastic Polymer Membranes with Tunable Properties for Gas Separation. Acs Appl. Mater. Interfaces.

[57] Guo M., Kanezashi M., Nagasawa H., Yu L., Yamamoto K., Gunji T., Ohshita J., Tsuru T. (2019). Tailoring the microstructure and permeation properties of bridged organosilica membranes via control of the bond angles. J. Membr. Sci..

[58] Scholes C.A., Stevens G.W., Kentish S.E. (2010). The effect of hydrogen sulfide, carbon monoxide and water on the performance of a PDMS membrane in carbon dioxide/nitrogen separation. J. Membr. Sci..

[59] Liu S., Liu G., Wei W., Xiangli F., Jin W. (2013). Ceramic Supported PDMS and PEGDA Composite Membranes for CO_2_ Separation. Chin. J. Chem. Eng..

